# Safety of a large language model-based clinical decision support system in African primary healthcare

**DOI:** 10.1038/s44360-026-00082-5

**Published:** 2026-03-10

**Authors:** Ambrose Agweyu, Paul Mwaniki, Wilkister Musau, Robert Korom, Lynda Isaaka, Conrad Wanyama, Sarah Kiptinness, Najib Adan, Mira Emmanuel-Fabula, Bilal A. Mateen

**Affiliations:** 1 KEMRI-Wellcome Trust Research Programme, Nairobi, Kenya; 2 Keprecon, Nairobi, Kenya; 3 London School of Hygiene and Tropical Medicine, London, UK; 4 PATH, Nairobi, Kenya; 5 Penda Health, Nairobi, Kenya; 6 FATH, Geneva, Switzerland; 7 University of Birmingham, Birmingham, UK; 8 PATH, London, UK

**Keywords:** Diagnosis, Health services

## Abstract

Here we conducted a retrospective evaluation of an electronic medical record-embedded large language model clinical decision support system deployed across 16 primary care clinics in Kenya, between July and September 2024. A panel of trained physicians reviewed 1,469 records. Hallucinations were uncommon, occurring in 50 encounters (3.4%, 95% confidence interval (CI) 2.5–4.5), and most often involved misexpanded acronyms or drug names. Clinical management guidance aligned with local guidelines in almost all cases (1,455; 99%, 95% CI 98.4–99.5). Despite this, clinicians did not modify documentation in 917 encounters (62%, 95% CI 59.9–64.9). Safety assessments identified actively harmful recommendations from the large language model in 115 encounters (7.8%, 95% CI 6.5–9.3), with 67 such recommendations appearing in the final documentation. Conversely, risk present in the clinician’s initial notes was fully mitigated in 118 encounters (8.0%, 95% CI 6.7–9.5 overall; 12.1%, 95% CI 9.5–15.2 of amended cases). Overall, the tool showed strong potential to support quality improvement, but the asymmetric adoption of harmful versus beneficial outputs underscores the need for usability optimization, local guardrails and prospective trials to confirm patient-level benefit.

## Abstract (web summary)

In a retrospective analysis of 1,469 patient encounters, a large language model-based clinical decision support system deployed across 16 outpatient clinics provided recommendations generally aligned with local guidelines, but with contrasting results on safety and relevance.

## Main

An adequately trained and equitably distributed health workforce is essential for delivering high-quality care and averting preventable morbidity and mortality^1^. However, many countries continue to face staffing shortages. The shortfall is most acute in sub-Saharan Africa, where reliance on cadres with short training pathways, high turnover and sustained outmigration undermines service delivery^2^. These workforce limitations give rise to wide disparities in quality of care across settings and are compounded by frontline providers, particularly in marginalized areas, lacking access to structured clinical mentorship that might have otherwise mitigated the impact on care^3^. Large language model (LLM)-based clinical decision support systems (CDSS) have the potential to help bridge these gaps by providing frontline clinicians with real-time, specialist physician-grade, contextually relevant diagnostic and management suggestions, potentially reducing clinically important errors, avoiding unnecessary treatments and minimizing delays in care by supporting appropriate and timely referrals^4,5^.

Although evidence of the safety and contextual appropriateness of LLM-based CDSS in low-resource primary healthcare settings remains scarce^6^, several studies in high-income countries have demonstrated the potential of this technology to improve diagnostic reasoning amongst primary care providers^7,8^, support treatment planning^9^ and even deliver therapeutic interventions^10^. However, evidence from well-resourced settings is of limited value to decision-makers in low- and middle-income countries (LMICs) because, as previous reports have noted^11–14^, many of these models are trained predominantly on data that fail to account for local epidemiology, resource availability and cultural context, reducing their reliability in LMIC healthcare settings. In the rare examples of proprietary models performing reasonably well on LMIC-based benchmarking datasets^15^, the limited transparency into the underlying training data undermines the ability to draw meaningful insights into the true generalizability of the tool across settings, and there is no consensus on whether these in silico evaluations provide any actual ‘guarantees’ around real-world performance^16^. In both cases, there is a very real risk wherein ‘naive’ application of an LLM to a novel low-resource setting means that errors arising from the aforementioned biases in the training data might lead to flawed clinical reasoning or guidance and potentially compromise patient safety if taken at face value^17^. Moreover, a recent user-experience research study in Kenya highlighted how this issue of lack of local grounding or outputs impacts frontline health worker perceptions of artificial intelligence (AI)-based solutions, with clinicians reporting that poorly contextualized responses (for example, suggesting a drug that was either unavailable or not part of the local protocol) undermined their confidence in the technology^18^. Recognizing these risks, the World Health Organization has emphasized that, while AI could help ‘bridge gaps’ in care for underserved communities, robust safeguards—such as local, real-world evaluations before deployment at scale—are needed to minimize risks and ensure ethical, context-appropriate use^19^.

**Fig. 1:**
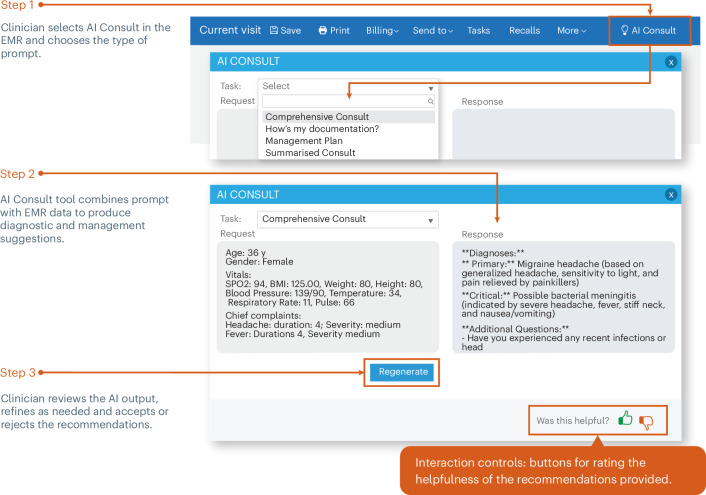
A summary of the AI Consult V1 workflow. Illustration of the GenAI-enabled workflow (referred to as AI Consult V1) that clinicians had access to during the study period. In summary, a button inside the EMR workflow allowed clinicians to select from one of four system-level prompts, which would be appended with all de-identified clinical data from the visit to create a query: • Comprehensive Consult: This prompt provided several paragraphs of feedback regarding differential diagnosis, diagnostic testing, history and physical examination gaps, and guideline-based treatments. Only encounters using this prompt were assessed in this study (see Supplementary Box 1 for the full system prompt). • Summarized Consult: The prompt provided no more than a paragraph of feedback, focusing on actionable steps a clinician could take to improve the quality of care. • Management Plan: The prompt provided a management plan for the condition without focusing on other aspects of care. • How’s my documentation?: This prompt provided a rating and constructive feedback points based on whether the clinical notes documented were adequate for the condition at hand. Notably, it was introduced only in September 2024. The prompt and EMR data (that is, the query) would flow to the OpenAI API, ChatGPT 4o, and return structured feedback to the clinician in a text box in real time. Clinicians then had the option to either disregard the LLM output or consider it when making their clinical decisions. Clinical officers in the study retained full authority for their clinical decisions throughout the study. This figure and elements of the caption were originally published in ref. ^18^, and reused under the associated CC-BY licence provisions, without any amendments.

We sought to evaluate the quality and safety of LLM-generated clinical advice and its influence on clinician decision-making in the context of a network of 16 outpatient facilities operated by Penda Health, a private social enterprise serving urban communities in Nairobi and Kiambu counties, Kenya. Penda Health operates Level 3a and 3b facilities, which are 24/7 outpatient urgent and primary care centres staffed primarily by clinical officers (non-physician healthcare providers licensed to independently diagnose, prescribe, and manage a wide range of outpatient conditions). All of Penda Health’s facilities utilize a cloud-based electronic health record system (developed by Easy Clinic (India)), into which an LLM-based CDSS (Fig. 1) was integrated and made available to staff.

## Results

The AI-enabled CDSS was used in 36,670 (46.8%) of the 78,366 clinical consultations at the 16 Penda Health facilities over the 3-month study period. Uptake increased over the evaluation period, although with substantial variation across clinics. In July 2024, consultations using the tool ranged from 29.3% (Zimmerman) to 53.7% (Kawangware). By September 2024, usage had increased across nearly all clinics, with 7/16 exceeding 60% utilization. The highest proportions were observed in Kangemi (80.6%), and Kasarani (75.1%). Clinics such as Zimmerman and Umoja 2 showed a more modest increase (reaching 44.7% and 40.7%, respectively, by September) (Supplementary Fig. 1).

### Study cohort characteristics

A total of 1,469 patient encounters were included in the evaluation, representing 1.9% of the clinical consultations over the evaluation period. Approximately a quarter of the patients were below 5 years of age (*n* = 350), 15% were aged 5–17 years (*n* = 225), half were aged 18–39 years (*n* = 730), 10% were aged 40–59 years (*n* = 150) and 1% were 60 years and above (*n* = 14). Based on the gender recorded in the electronic medical record (EMR), 612 (42%) were male and 857 (58%) were female. Key results stratified by patient age group are presented in Table 1.

**Table 1:** Selected evaluation metrics across all domains of quality and safety, stratified by patient age

	Overall	0–4	5–17	18–39	40–59	60+
	*N* = 1,469	*N* = 350	*N* = 225	*N* = 730	*N* = 150	*N* = 14
Quality of initial clinical documentation (Q4)						
‘Totally inadequate’ OR ‘Needs significant improvement’	341 (23%, 21–25)	72 (21%, 16–25)	45 (20%, 15–26)	178 (24%, 21–28)	41 (27%, 20–35)	5 (36%, 13–65)
‘Acceptable’ OR ‘High quality’	1,128 (77%, 75–79)	278 (79%, 75–84%)	180 (80%, 74–85)	552 (76%, 72–79)	109 (73%, 65–80)	9 (64%, 35–87)
Safety concerns associated with initial documentation						
No safety concern identified (Q6)	922 (63%, 60–65)	212 (61%, 55–66)	130 (58%, 51–64)	490 (67%, 64–71)	82 (55%, 46–63)	8 (57%, 29–82)
‘Mild’ OR ‘Moderate’ concern identified (Q10)	410 (28%, 26–30)	114 (33%, 28–38)	82 (36%, 30–43)	164 (22%, 19–26)	46 (31%, 23–39)	4 (29%, 8–58)
‘Severe’ OR ‘Life-threatening’ risk identified (Q10)	137 (9%, 8–11)	24 (7%, 4–10)	13 (6%, 3–10)	76 (10%, 8–13)	22 (15%, 9–21)	2 (14%, 2–43)
LLM addressed clinical issues identified in initial documentation^1^ (Q11)	1,419 (97%, 96–97)	343 (98%, 96–99)	215 (96%, 92–98)	700 (96%, 94–97)	147 (98%, 94–100)	14 (100%, 77–100)
LLM prioritized advice appropriately based on documentation^2^ (Q12)	1,456 (99%, 98–100)	349 (100%, 98–100)	224 (100%, 98–100)	720 (99%, 97–99)	149 (99%, 96–100)	14 (100%, 77–100)
LLM response included hallucinations (Q3)	50 (3%, 3–4)	10 (3%, 1–5)	14 (6%, 3–10)	21 (3%, 2–4)	5 (3%, 1–8)	0 (0%, 0–0)
LLM communication quality was ‘Adequate’ OR ‘Effective’ (Q14)	1,459 (99%, 99–100)	349 (100%, 98–100)	224 (100%, 98–100)	723 (99%, 98–100)	149 (99%, 96–100)	14 (100%, 77–100)
LLM provided novel diagnostic insights^3^ (Q18)	1,384 (94%, 93–95)	329 (94%, 91–96)	207 (92%, 88–95)	689 (94%, 92–96)	145 (97%, 92–99)	14 (100%, 77–100)
LLM response aligned with local clinical guidelines^4^ (Q21)	1,455 (99%, 98–99)	347 (99%, 98–100)	223 (99%, 97–100)	724 (99%, 98–100)	147 (98%, 94–100)	14 (100%, 77–100)
LLM provided novel management-related insights^5^ (Q22)	1,461 (99%, 99–100)	350 (100%, 99–100%)	222 (99%, 96–100%)	725 (99%, 98–100)	150 (100%, 98–100)	14 (100%, 77–100)
Appropriateness of LLM response in patient’s socioeconomic/cultural context or resource limitations (Q23)						
AI attempted to, unsuccessfully, adapt to patient’s context	2 (0%, 0–0)	0 (0%, 0–0)	1 (0%, 0–2)	1 (0%, 0–1)	0 (0%, 0–0)	0 (0%, 0–0)
AI failed to acknowledge or did not attempt to adapt to context	2 (0%, 0–0)	1 (0%, 0–2)	0 (0%, 0–0)	1 (0%, 0–1)	0 (0%, 0–0)	0 (0%, 0–0)
Response was not culturally/contextually appropriate	3 (0%, 0–1)	2 (1%, 0–2)	1 (0%, 0–2)	0 (0%, 0–0)	0 (0%, 0–0)	0 (0%, 0–0)
No limitations/constraints described requiring adapted response	1,153 (78%, 76–81)	281 (80%, 76–84)	168 (75%, 68–80)	579 (79%, 76–82)	116 (77%, 70–84)	9 (64%, 35–87)
Yes, AI adapted response fully to context	309 (21%, 19–23)	66 (19%, 15–23)	55 (24%, 19–31)	149 (20%, 18–24)	34 (23%, 16–30)	5 (36%, 13–65)
LLM response contains actively harmful recommendations^6^ (Q24)	115 (8%, 7–9)	24 (7%, 4–10)	20 (9%, 6–13)	56 (8%, 6–10)	15 (10%, 6–16)	0 (0%, 0–0)
Initial documentation risk mitigated by LLM (Q28)						
No	548 (37%, 35–40)	141 (40%, 35–46)	96 (43%, 36–49)	240 (33%, 29–36)	65 (43%, 35–52)	6 (43%, 18–71)
No risk was identified in the initial documentation	803 (55%, 52–57)	182 (52%, 47–57)	110 (49%, 42–56)	431 (59%, 55–63)	73 (49%, 40–57)	7 (50%, 23–77)
Yes (Fully OR Partially)	118 (8%, 7–10)	27 (8%, 5–11)	19 (8%, 5–13)	59 (8%, 6–10)	12 (8%, 4–14)	1 (7%, 0–34)
Harmful LLM output reflected in final documentation^7^ (Q29)	67 (5%, 4–6)	16 (5%, 3–7)	13 (6%, 3–10)	25 (3%, 2–5)	13 (9%, 5–14)	0 (0%, 0–0)
LLM response was helpful (Q30)	1,256 (86%, 84–87)	307 (88%, 84–91)	189 (84%, 79–89)	617 (85%, 82–87)	131 (87%, 81–92)	12 (86%, 57–98)

Values shown as *n* (%, 95% CI). (1) Combines the ‘Entirely’ and ‘Mostly’ responses to question 11. (2) Combines the ‘Entirely’ and ‘Partly’ responses to question 12. (3) Combines the ‘Yes’ and ‘Somewhat’ responses to question 18. (4) Combines the ‘Yes’ and ‘Mostly’ responses to question 21. (5) Combines the ‘Yes’ and ‘Somewhat’ responses to question 22. (6) Combines the ‘major’ and ‘minor’ safety concerns responses to question 24. (7) Applicable in observations where LLM provided actively harmful response, and combines the ‘Yes – Fully’ and ‘Yes – Partially’ responses to question 29.

Respiratory system presentations were the most common (*n* = 561; 38%), followed by gastrointestinal (*n* = 362; 25%) and then genitourinary/reproductive (*n* = 217; 15%) presentations. Other categories included dermatological (*n* = 149; 10%), musculoskeletal (*n* = 130; 9%), febrile/infectious (*n* = 109; 7%), neurological/psychiatric (*n* = 73; 5%), ear, nose and throat/dental/ophthalmological (*n* = 64; 4%), cardiovascular (*n* = 39; 3%) and unspecified/other (*n* = 101; 7%).

### Baseline assessment of the clinicians’ input (to the AI Consult) documentation

Initial clinical documentation was rated as acceptable for 878/1469 records (60%, 95% confidence interval (CI) 57.2–62.9) while a further 250 (17%, 95% CI 15.1–19.0) were graded as high quality. Documentation requiring significant improvement accounted for 279 cases (19%, 95% CI 17.0–21.1), and 62 records (4%, 95% CI 3.3–5.4) were rated as totally inadequate. Interrater reliability statistics for all metrics are reported in Supplementary Table 1.

**Fig. 2:**
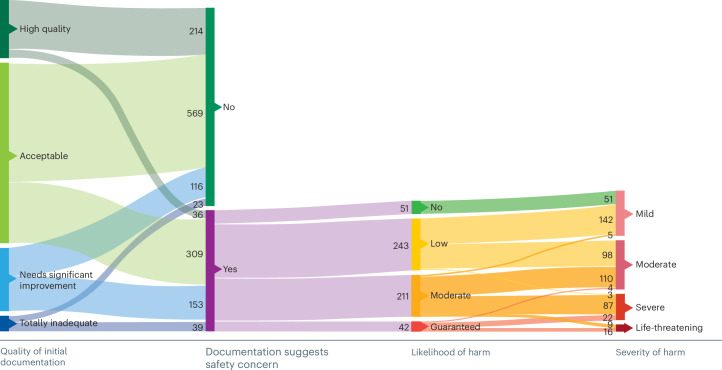
Sankey plot of documentation quality, associated risk, likelihood, severity and type of harm. Illustration of flows from the quality of initial clinical documentation (high quality, acceptable, needs significant improvement or totally inadequate) to the presence of identified safety concerns, the estimated likelihood of harm (none, low, moderate or guaranteed) and the corresponding severity of harm (mild, moderate, severe or life-threatening). Flow widths are proportional to the number of encounters.

Safety concerns were identified in the initial documentation of 547 records (37%, 95% CI 34.8–39.8). The most common safety concerns were inappropriate medication in 272 cases (49%, 95% CI 45.5–54.0), omission of critical differential diagnoses in 230 (42%, 95% CI 37.9–46.3) and incorrect diagnoses in 120 cases (22%, 95% CI 18.5–25.6). Less frequent issues included incorrect or unsafe dosing (61; 11.2%, 95% CI 8.6–14.1), inappropriate investigations or tests (46; 8.4%, 95% CI 6.2–11.1), culturally or contextually inappropriate guidance (5; 0.9%, 95% CI 0.3–2.1) and other concerns (66; 12.1%, 95% CI 9.5–15.1). The perceived likelihood of harm associated with these concerns varied considerably: low in 243 cases (44%, 95% CI 40.2–48.7), moderate in 211 cases (39%, 95% CI 34.5–42.8), guaranteed in 42 cases (7.7%, 95% CI 5.6–10.2) and none in 51 cases (9.3%, 95% CI 7.0–12.1). Similarly, the severity of potential harm was rated as moderate in 212 cases (39%, 95% CI 34.7–42.9), mild in 198 (36%, 95% CI 32.2–40.4), severe in 112 (20%, 95% CI 17.2–24.1) and life-threatening in 25 (4.6%, 95% CI 3.0–6.7). Safety concerns were less common when initial documentation was high quality and rose as quality declined. Specifically, 36/250 high-quality notes (14%, 95% CI 10.3–19.4) contained safety concerns, compared 309/878 acceptable notes (35%, 95% CI 32.0–38.5), 163/279 records needing significant improvement (58%, 95% CI 52.4–64.3) and 39/62 totally inadequate records (63%, 95% CI 49.7–74.8). In lower-quality strata, concerns also shifted towards higher likelihood and severity categories. Summaries of the likelihood, severity and type of harm, stratified by each documentation quality rating, are presented in Supplementary Tables 2–6 and illustrated in Fig. 2.

### Appropriateness of the LLM-based CDSS outputs

#### Global assessment

Of the 1,469 AI-generated responses, 1,213 (83%, 95% CI 80.5–84.5) fully addressed the clinical issues identified in the initial documentation, with a further 206 (14%, 95% CI 12.3–15.9) mostly addressing them. Only 49 responses (3.3%, 95% CI 2.5–4.4) partially addressed the issues, and a single response (<0.1%, 95% CI 0.0–0.4) was judged to have no relevance to the clinical problem. Nearly all AI-generated responses, 1,456.1469 (99%, 95% CI 98.5–99.5) prioritized the advice appropriately based on the initial documentation, and 1,459 responses (99% 95% CI 98.8–99.7) were assessed as having effectively communicated their advice.

Hallucinated content, defined as fabricated or clinically inaccurate information, was identified in 50 responses (3.4%, 95% CI 2.5–4.5). Post-hoc qualitative review of evaluators’ comments grouped hallucinations into the following four themes: misidentified drugs or misexpanded acronyms (*n* = 14) (for example, ‘FGC’ read as ‘female genital circumcision’ rather than fair general condition); contradictions of the initial documentation (*n* = 8) (for example, stating there were no signs of dehydration when the clinical note did not document hydration status); clinical parameter misinterpretation (*n* = 8) (for example, flagging SpO_2_ 95% at Nairobi’s altitude ~1,800 m as abnormal); and cases in which the rationale for the hallucination was insufficiently specified (*n* = 20).

#### Diagnostic reasoning

The presence of any diagnostic reasoning by the LLM was noted in nearly all outputs (1,443; 98%, 95% CI 97.4–98.8). Regarding the quality of differential diagnosis, 1,220 (83%, 95% CI 81.0–84.9) of responses demonstrated strong and well-reasoned consideration of alternatives (or rightly affirmed that the clinician’s differential was appropriate), while 223 (15%, 95% CI 13.4–17.1) had partial or incomplete differential reasoning (for example, demonstrated by a 5-year-old child with a month-long history of rhinorrhea and snoring, focusing on sinusitis management without considering adenoidal hypertrophy or other obstructive etiologies). A small number of responses (24; 1.6%, 95% CI 1.0–2.4) contained major reasoning gaps or misleading information such as inappropriate reassurance about granulocytosis or missing pregnancy testing in a 28-year-old woman with right lower quadrant pain, and only two responses (0.1%, 95% CI 0.0–0.5) were deemed irrelevant (that is, grossly misaligned with the input content). In terms of novelty (given that sometimes simply agreeing with the clinician’s differential was deemed a high-quality response), 1,384 AI-generated responses (94%, 95% CI 92.9–95.4) provided new diagnostic insights. For example, in a 25-year-old woman initially diagnosed only with acute rhinitis, the LLM identified unrecognized microcytic anaemia (based on abnormal hemogram indices) and suggested iron deficiency as a plausible comorbidity, which the clinician subsequently incorporated into the final documentation. In 85 encounters (5.8%, 95% CI 4.6–7.1) the LLM responses offered no novel insight or were misleading. For contextual relevance (for example, an awareness of the local epidemiology) in its diagnostic reasoning, 1,384 responses (94%, 95% CI 92.9–95.4) were fully aligned with the patient’s clinical and social context, with partial alignment observed in 78 cases (5.3%, 95% CI 4.2–6.6) and complete misalignment in just 7 cases (0.5%, 95% CI 0.2–1.0).

#### Clinical reasoning (investigations and treatment planning)

The vast majority of AI responses (1,460; 99%, 95% CI 98.8–99.7) provided clinical management advice (for example, examinations, tests, referrals and treatments). Almost all responses (1,455; 99%, 95% CI 98.4–99.5) aligned with local clinical guidelines, and 1,461 responses (99%, 95% CI 98.9–99.8) contributed additional management-related insights beyond the initial documentation. Appropriateness of the LLM response in the patient’s socioeconomic or cultural context or resource limitations was high with no conflict in 1,153 encounters (78%, 95% CI 76.3–80.6); fully adapted in 309 (21%, 95% CI 19.0–23.2); with only small numbers showing issues—2 attempted but failed to adapt (0.1%, 95% CI 0.0–0.5), 2 did not attempt to adapt (0.1%, 95% CI 0.0–0.5) or 3 not culturally/contextually appropriate (0.2%, 95% CI 0.0–0.6).

#### Safety concerns associated with LLM outputs

Overall, 115 responses (7.8%, 95% CI 6.5–9.3) included active recommendations that evaluators considered potentially harmful: 37 (2.5%, 95% CI 1.8–3.5) were regarded to have posed major safety concerns and 78 (5.3%, 95% CI 4.2–6.5%) minor concerns. Among these 115 responses, evaluators judged that, in 25 cases (22%,95% CI 14.6–30.4), the clinician appeared to have fully adopted the harmful advice, and in 42 cases (37%, 95% CI 27.7–46.0) partially adopted it. In 48 cases (42%, 95% CI 32.6–51.3), the harmful recommendations were not acted upon. The types of issues identified in the subset of potentially harmful AI responses were broadly similar to those found in initial clinical documentation. Follow-up reviews confirmed no resulting adverse patient outcomes.

The most common potentially harmful LLM outputs were inappropriate medication recommendations noted in 54 cases (46.2%, 95% CI 36.9–55.7) and omission of critical differential diagnoses in 37 cases (31.6%, 95% CI 23.3–40.9). Incorrect diagnoses were noted in 17 responses (14.8%, 95% CI 9.0–22.3), while less frequent issues included incorrect or unsafe dosing in 6 cases (5.2%, 95% CI 1.9–11.0), inappropriate investigations in 3 (2.6%, 95% CI 0.5–7.4), culturally/contextually inappropriate guidance in 3 (2.6%, 95% CI 0.5–7.4) and other concerns in 24 cases (20.9%, 95% CI 13.9–29.2).

### Clinician response to LLM recommendations and impact on patient safety

In most encounters (917; 62%, 95% CI 59.9–64.9), clinicians made no LLM-induced changes; 358 (24%, 95% CI 22.2–26.6) had minor LLM-induced changes. For example, a 2-year-old with a short history of cough, sneezing and runny nose was correctly diagnosed with a viral upper respiratory infection. The LLM did not alter the diagnosis or overall management but made small documentation refinements, including adjusting the cetirizine dose to an age-appropriate level. In 194 encounters (13%, 95% CI 11.5–15.0), the LLM induced major changes. For example, in a child initially assessed for cough and abdominal pain, the original documentation lacked any bowel-related diagnosis; after the LLM highlighted chronic faecal soiling and the possibility of functional constipation, the clinician added a diagnosis of encopresis, performed an anal examination and instituted a full management plan including laxatives, dietary advice and a toileting routine. Among the 552 encounters with any change, edits most often concerned the treatment plan (436; 79%, 95% CI 75.3–82.3), followed by the differential diagnosis (184; 33%, 95% CI 29.4–37.4), history or examination findings (131; 24%, 95% CI 20.2–27.5) and the follow-up plan (129; 23%, 95% CI 19.9–27.1); investigations were revised in 98 (18%, 95% CI 14.7–21.2), and other edits occurred in 18 (3%, 95% CI 1.9–5.1). Of these 552 encounters, the LLM fully mitigated a potential harm in 67 (12.1%, 95% CI 9.5–15.2) and partially mitigated risk in 51 (9.2%, 95% CI 7.0–12.0); the remaining 434 (78.6%, 95% CI 75.0–82.0) either had no initial risk identified or showed no mitigation.

**Fig. 3:**
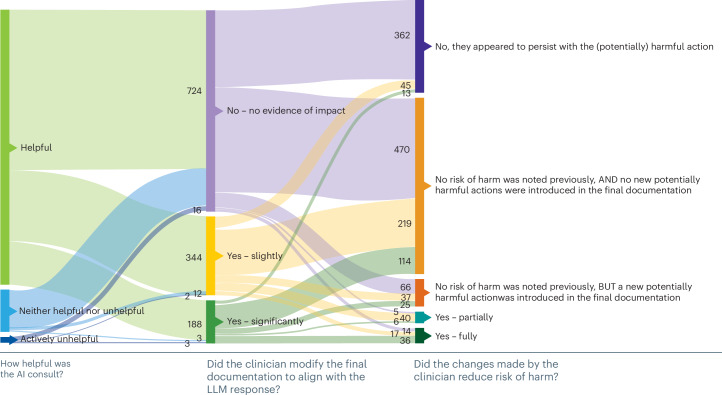
Sankey plot of modifications to final clinical documentation, relationship to LLM outputs and implications for patient safety. Linkage between LLM helpfulness ratings and clinician modifications and resulting safety outcomes. Flow widths are proportional to encounter counts and illustrate that many consultations showed no change, while beneficial and harmful recommendations were differentially adopted.

**Fig. 4:**
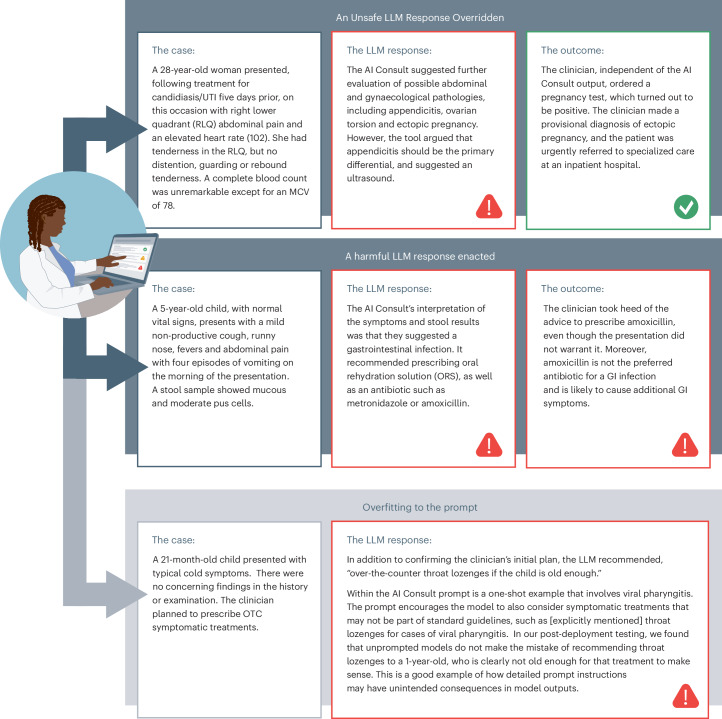
Summaries of real-world examples of AI Consult impacting (or not) care quality (both positively and negatively). Three real-world cases demonstrate different patterns of LLM influence on care. Top: a potentially unsafe LLM recommendation was appropriately overridden by the clinician, resulting in correct diagnosis and referral. Middle: a harmful recommendation was followed, leading to inappropriate antibiotic prescribing. Bottom: an example of prompt overfitting, where model outputs reflected prompt wording rather than clinical necessity. GI, gastro-intestinal; OTC, over the counter; UTI, urinary tract infection.

In 803 encounters (55%, 95% CI 52.1–57.2), no risk was initially identified, and no new safety concerns were introduced. However, in 420 cases (29%, 95% CI 26.3–31.0), clinicians continued with the potentially harmful course of action identified in the initial documentation, despite the LLM explicitly providing helpful guidance in 362 cases (25%, 95% CI 22.5–26.9). Apart from the aforementioned 115 cases of LLM-induced harmful action, there were another 13 cases (0.9%, 95% CI 0.5–1.5) of spontaneous (not LLM-induced) changes to the final documentation that introduced a risk to the patient (which were not present in the initial documentation) (Fig. 3). Figure 4 highlights three (de-identified) real-world examples of LLM-related harm introduction.

### Inference costs

The total cost of running the LLM-based CDSS for the 1,469 encounters in this study was US$7.81, which translates to an average of 0.5 (US) cents, assuming one application programming interface (API) call per encounter with an average of 670 input tokens and 364 output tokens.

## Discussion

In this real-world evaluation of an LLM-based CDSS in Kenyan primary care clinics, we found that the system’s suggestions were largely (clinically) well reasoned, appropriate to the local context, effectively communicated and with only a very small number of ‘hallucinations’ (a large number of which were incidental misunderstandings of uncommon acronyms)—all notable given that the underlying system was an off-the-shelf LLM (that is, not purpose-built, not fine-tuned and not augmented with contextually relevant information using a retrieval-augmented generation framework). However, in nearly two-thirds of encounters where the LLM was utilized (*n* = 917, 62%), there was no observable impact on clinician decision-making, suggesting that there is an important difference between interaction/engagement with such tools and incorporation of the outputs. Critically, of these encounters, 362 were instances where the LLM provided beneficial guidance, but the clinician did not heed it, illustrating the unrealized potential of this technology. In instances where the LLM’s advice was acted on (*n* = 552), approximately twice as many instances of beneficial (to the patient) (*n* = 118) recommendations were followed compared with harmful recommendations (*n* = 67). Notably, however, this represents 22% of the instances in which beneficial advice was provided, and 58% of the harmful outputs, suggesting that harmful guidance was much more likely to be acted upon by clinicians. Although the proportion of total encounters (7.8%) that these harmful recommendations reflect is relatively low, it underscores the genuine risks associated with integrating LLMs into care. In summary, the results of the study provide compelling evidence for the potential of LLM-based CDSS to improve the quality of care in low-resource settings, but further research is required to optimize the user experience and utilization of the system outputs, create effective safeguards to reduce the risk of (uncommon) harmful outputs and confirm the patient-level impact via a controlled, interventional study^20^.

Advanced LLMs have shown impressive capabilities on medical examinations^21,22^ and standardized case vignettes^23^, often matching or surpassing human clinicians on structured clinical problem-solving tasks. This has led to a proliferation of EMR-integrated solutions that leverage these purported clinical reasoning capabilities^24,25^; however, no other studies appear to report a clinical evaluation of a real-world implementation of an EMR-embedded, CDSS-style copilot, regardless of context, making it difficult to determine whether the results of this study align with expectations. The closest analogue is Google’s objective structured clinical examination-based (simulated-patient-based) assessment of G-AMIE^9^, which is an autonomous multi-agent system for information solicitation (the major difference from the AI Consult tool in this study), diagnostic reasoning and clinical management planning. G-AMIE, based on Google’s Gemini 2.0 Flash, can identify the most appropriate diagnosis and treatment plan in most cases, similar to the abilities observed for AI Consult in this study. Notably, less extensively trained clinicians (nurse practitioners and physician associates), when augmented by the system, appeared to perform better than more extensively trained primary care physicians, largely because the latter group showed higher confidence in their diagnoses and treatment plans despite performing no better, and in some instances worse. In essence, the issue of (unjustified) confidence appears to be a consistent theme in limiting the potential (beneficial) impact of AI-based tools in improving quality of primary care.

A recent systematic review noted that many AI healthcare solutions in LMICs remain at the proof-of-concept stage and fully implemented real-world evaluations in low-resource settings are rare, limiting our understanding of their true impact and cost-effectiveness^14^. Of the limited high-quality evidence available from LMICs (randomized controlled trials), we identified only one economic evaluation of an AI-based tool^26,27^, and it suggests that the technology is not cost-effective^28^. Using malaria as a comparator, we can put the reported costs of this solution into context (which excludes non-token expenses: engineering/integration, infrastructure, retries, security/compliance, storage, monitoring and so on); the willingness to pay in low-resource settings for a rapid malaria diagnostic test is 20–50 (US) cents^29,30^, and for drug therapy is up to US$2^30^. In essence, the LLM-based CDSS costs are considerably lower than what health systems and individuals (that is, out-of-pocket expenses) can afford for locally relevant, priority health issues. Although we cannot compare efficacy or cost–benefit statistics across the two sets of interventions based on the results of this study, the absolute cost of the proposed AI-based intervention does not appear to be prohibitively large.

As noted earlier, previous reports have cautioned that the deployment of AI models trained predominantly on data from high-income countries may fail to account for local disease epidemiology, resource availability and cultural practices, thereby limiting their reliability in LMIC environments^12–14^. In this study, we observed confirmatory evidence of this concern: when the AI’s recommendations were inappropriate, it was often because they were not well tailored to the Kenyan primary care context—for example, suggesting diagnostic tests unavailable in these clinics or medications not included on local formularies. Assessing solely the absolute number of such events might lead one to believe that this issue is not actually that much of a problem, given that it is substantially smaller than the number of cases in which beneficial recommendations were provided.

However, overall trust in the system must be sufficient to ensure that clinicians are responsive to the AI’s advice when it truly matters. Gaining that trust requires consistent demonstration of accuracy and value^31^. As such, the indirect impact of this minority of cases is that many of the beneficial recommendations were ignored; this was confirmed by a parallel user-experience study conducted with the clinicians at Penda Health, who reported a loss of confidence in the tool when it provided unaligned responses^18^.

A major strength of this study is the multi-site design. By evaluating the CDSS across 16 clinics, we captured how the AI performs in routine urban primary care practice. This breadth improves the relevance and generalizability of our findings within the Kenyan context, compared with simulations or single-site pilots. The study’s evaluation methods engaged an independent panel of experienced local physicians to review cases, with a standardized training and calibration process to improve consistency in their judgements. Multiple reviewers assessed each case domain, and we conducted interrater reliability checks to quantify consistency. In addition, our sample size is substantially larger than most prior evaluations of AI in healthcare in LMICs, affording more precise estimates of AI performance. Finally, the CDSS was evaluated within the live EMR system, reflecting real clinician–AI interactions with minimal Hawthorne effect, and thus probably capturing authentic behaviour under normal time pressure and responsibility.

There are several limitations to this study. First, the observational, retrospective design precludes any definitive conclusions about causality or clinical impact; for example, we cannot definitively state that any changes made are a consequence of the LLM as we do not know what was in the clinician’s mind, but at the time of consulting the LLM had not yet been documented. Moreover, we did not have a contemporaneous control group of encounters without AI usage, so we cannot quantitatively determine how much the AI actually improved diagnostic accuracy, treatment appropriateness or efficiency compared with standard care. Any improvements in error rates are based on before-and-after comparisons within the same encounter rather than a randomized comparison across encounters. There is a risk of confounding in cases where clinicians may have selectively used the AI on more complex cases, meaning the overall error profile with AI could differ from that of typical cases for reasons unrelated to the tool itself. A prospective controlled trial is currently underway in Kenya, and its results are expected to provide high-quality evidence on outcomes such as treatment success rates and safety events under LLM-assisted care^6,20^. Moreover, our evaluation relied on subjective expert review of documentation, introducing some measurement uncertainty. Assessing whether a diagnosis or treatment was appropriate often requires clinical judgement, and experts may differ, especially when documentation is limited. We sought to minimize bias through rater training, explicit criteria and independent review with adjudication; however, some variability persisted. Notably, of the 37 cases categorized as major safety concerns, 10 were contributed by a single evaluator. In a post-study audit of cases flagged as major, many reflected judgement calls rather than unequivocal errors; most involved omissions (for example, endorsing an incorrect investigation or treatment already present in the clinician’s initial documentation) rather than the LLM actively recommending a harmful action (Supplementary Material 3). Furthermore, we evaluated a potentially suboptimal configuration, as the LLM was neither formally localized (for example, through fine-tuning) nor explicitly augmented with local Kenyan clinical guidelines. Despite its reasonably robust performance, it is more likely than not that localization could have yielded additional performance gains. Finally, the evaluation covered a relatively short time window (3 months) and a specific version of the LLM. AI models continue to evolve rapidly, and user behaviour also shifts over time. Thus, our snapshot may not capture long-term performance trends, and specifically, the impact of any training effects from repeated nudging.

Several additional avenues of exploration would benefit future implementation programmes and product development exercises. Apart from trust, there are probably other factors influencing the suboptimal utilization of LLM outputs, which, if identified, might allow for a better construction of human–computer interaction to increase the uptake of beneficial recommendations and thus improve quality of care. Moreover, the higher likelihood of harmful recommendations being followed than beneficial ones is a related, but equally important, question worthy of further investigation to isolate the cause. Furthermore, future studies might consider exploring different deployment models, such as LLM advice only for certain conditions, only visible to supervising physicians or limited to serving as training tools and uncoupled from individual clinical interactions, to determine what yields the best balance of benefit and safety.

This study highlights the dependency between technological fixes and systemic infrastructure. The setting of this study was a private, reasonably well-resourced primary care network in an urban African context. The clinicians at Penda Health are supported by a robust cloud-based digital infrastructure and regular training, and have access to relatively comprehensive supplies, laboratories and imaging capacity. It is this investment in the enabling environment and underlying systems (for example, supply chains, referral networks and training programmes) that meant clinicians could act on the outputs of the AI-based solution. Outcomes and LLM utilization patterns will undoubtedly differ in settings with fewer resources, different patient demographics or alternative healthcare workflows^32^. Caution is therefore warranted when extrapolating our results to dissimilar environments; however, this also reflects a broader lesson: technology can only be as effective as the environment in which it operates. Reports from other LMIC deployments have documented practical barriers such as poor internet connectivity, lack of device integration, low digital literacy and poor integration into clinical workflow, limiting the usefulness of AI systems^33,34^. Our findings reinforce, rather than reduce, the need to address these concerns to ensure equitable benefits.

There are also open questions surrounding the regulatory status of LLM-based technologies in healthcare settings^35^. Continuous post-deployment monitoring and evaluation of AI systems should be standard practice, but without a regulatory requirement, it is unclear why developers and deployers would agree to this additional burden. Ongoing surveillance is particularly important given that proprietary AI models are often updated, and performance of any AI tool can drift^36^. As such, health systems deploying AI should plan for this kind of life-cycle monitoring, similar to pharmacovigilance for new drugs or post-market device surveillance^37^. The current lack of clarity creates substantial inertia and risk associated with progressing products that could be classed as a medical device towards deployment at scale, limiting the ability to realize the benefits of this technology.

Our findings indicate that EMR-embedded, LLM-based decision support systems have strong potential to enhance clinical decision-making in primary care in sub-Saharan Africa, offering relevant and contextually appropriate guidance in many scenarios. Ongoing clinical trials should confirm whether the observed benefits translate into patient-level impact. Regardless, further research is needed to fully understand how clinicians interact with AI-based CDSS, including when they choose to consult it, how they interpret its advice and why they sometimes override or ignore it. It appears that, at a minimum, localizing AI systems—through training on region-specific data and incorporating national treatment guidelines—will be essential for building trust in AI-assisted care, thereby maximizing uptake and the overall impact of this technology. In parallel, interdisciplinary research on governance, ethics and policy must keep pace with technical advances if we are to fully and equitably unlock the benefits of this technology.

## Methods

### Study design and setting

We conducted a retrospective observational study using routinely collected electronic health record data from primary healthcare facilities in Kenya. The study analysed data collected at 16 clinics operated by Penda Health, a private social enterprise and primary care network serving urban communities in Nairobi and Kiambu counties. In early 2024, these clinics implemented an LLM-based CDSS integrated into their EMR (described in more detail below and illustrated in Fig. 1). The system allows clinicians to click an ‘AI Consult’ button during patient encounters, which prompts the LLM (specifically GPT-4o) to provide actionable diagnostic and therapeutic guidance tailored to the case.

### Participants and data collection

We included patient encounters from a 3-month inclusion period (1 July 2024 to 30 September 2024) during which the AI Consult feature was actively used in clinical workflows. Eligible encounters were those in which the attending clinician engaged the AI Consult for the patient’s case using the comprehensive consult prompt. From all such encounters during the period, we drew a proportionally allocated, age-stratified random sample of 1,500 cases (strata: 0–4, 5–17, 18–39, 40–59, 60+) to preserve the marginal age distribution of the source dataset. This sample was selected for detailed review. The sample size of 1,500 cases was chosen pragmatically to balance reviewer workload with precision and subgroup coverage, and is sufficient to estimate proportions as low as 2% with ±1% precision, accounting for a design effect of 2. Encounters were excluded if the patient had previously opted out of research using their data (in line with the clinic network’s consent policies), and any excluded record was replaced with another randomly selected case from the same period.

All data originated from Penda Health’s EMR system and were extracted from an Azure SQL Server database, which maintains a consolidated replica of the EMR data, separate from the operational system. For each selected encounter, we retrieved de-identified clinical data (patient demographics, presenting complaints, medical history, examination findings, diagnoses, treatments and follow-up outcomes) alongside the full transcript of the LLM’s consultation (including the input data and the LLM’s output text). A unique study ID was assigned to each record, and any personal identifiers were removed before analysis to ensure confidentiality. Data extraction and de-identification were performed by authorized data managers using automated scripts within the secure hospital data environment. No patient contact was required, and all data remained within secure servers during the evaluation.

### LLM CDSS description

The LLM-based CDSS implemented throughout Penda Health facilities that were evaluated in this study provided decision support by analysing the patient’s clinical notes and suggesting possible diagnoses, investigations and management steps. The system operates by the click of a button, where a prompt instructs the LLM to consider the patient’s symptoms, vital signs and clinical history (entered by the clinician into the EMR and including any documented physical examination and tests), to generate a consultative response as if advising a primary care physician in an urgent care setting. For example, the LLM output might include a differential diagnosis list, recommendations for additional tests or referrals, and treatment or counselling suggestions. Clinicians could review this output in real time during the consultation and decide whether to follow or ignore the advice. The LLM (GPT-4o) is a generative model that was pretrained on a broad medical knowledge base and underwent local testing by the Penda Health clinical team; however, it was not specifically trained on Kenyan patient data before deployment. All clinicians received a focused orientation on how to use the tool and its limitations (for example, that it can produce errors) before its integration into practice. The present study evaluates the historical outputs of this tool in routine use, rather than introducing a new intervention. Notably, the AI Consult tool was first introduced in February 2024, alongside user training.

### Expert evaluation panel selection

Penda Health’s Human Resources department ran an open recruitment following a job advert posted on 30 January 2025 on the organization’s social media pages. A total of 162 applications were received. Eligibility targeted medical officers who had completed an internship in Kenya, were registered with the Kenya Medical Practitioners and Dentists Council, and were actively practicing for at least 1 h per week. Screening, including verification of current medical licences, excluded 116 applicants who did not meet minimum requirements, with 46 candidates advancing. These 46 candidates underwent a curriculum vitae review and a phone interview, after which 30 were selected and received offers. All selected evaluators underwent training on the research protocol and scoring criteria prior to reviewing study cases (see Supplementary Material 2 for the scoring criteria and guidance on completion). This training took the form of a half-day in-person workshop. It included a guided review of example cases (separate from the study sample).

### Calibration and reference standard

After the workshop, a calibration exercise was conducted, during which evaluators scored a set of 30 records (sampled from the 1,500 records). We excluded one routine follow-up visit, resulting in 1,469 for the formal evaluation. Four physicians had independently scored each of these 30 records (authors: A.A., R.K., S.K. and B.A.M.). The panel then held consensus meetings to establish a reference standard for each question. In most items, a single consensus response was reported because either all four reviewers selected it, a simple majority (three of four) selected it, or the team discussed and agreed to adopt it. In the rare cases where two responses were shown, the reviewers were evenly split between those two; only those two were considered valid, and the ±1 rule did not apply. The ±1 rule provided a narrow tolerance within the same sentiment band. For example, for the options (Yes (strong and well-reasoned); Yes (with some gaps); No (major gaps or misleading); No (irrelevant)) if the consensus was Yes (with some gaps), no No option was admissible under ±1. For three-level items (Yes; Yes (partial); No), if the consensus was No (not at all), no ±1 applied because it was the only valid negative. For four-level scales (Entirely; Mostly; Somewhat; Not at all), if the consensus was Mostly, only the adjacent middle option Somewhat was within ±1; and Entirely and Not at all were not. Yes/No questions always had a single valid response with no ±1 tolerance. For risk-of-harm items, consensus was defined with particular care. The ±1 tolerance was applied within severity ranges only (for example, movement from low to moderate or from severe to life-threatening) and was not applied across the low-to-severe divide. In sum, consensus lay within the sentiment of the agreed response, constrained to adjacent options where specified.

Only those evaluators who demonstrated acceptable agreement with reference standards (defined as no more than 50% discordant but within the ±1 range, as well as no scores two points or more from the reference standard) during this calibration phase were permitted to proceed to rate additional study records, to maintain high interrater reliability. Notably, all evaluators passed the calibration exercise.

### Evaluation procedure

From 1,470 cases in the evaluation dataset, we excluded one routine follow-up visit, leaving 1,469 encounters for formal evaluation. There were a total of 1,880 LLM-generated responses for the 1,469 clinical encounters, indicating that, in a minority of cases, the AI Consult tool was used more than once in a clinical encounter. In this study, we only consider the initial AI Consult response in relation to the case. Following calibration, the evaluation panel independently reviewed each LLM-assisted encounter. Cases were grouped into batches of 30, with a maximum of 6 batches per evaluator to limit fatigue and potential scoring bias; each evaluator reviewed 2–4 batches.

Each record in the study sample was reviewed by at least one evaluator. To monitor quality and reliability, a random 10% subset of the cases was independently evaluated by a second panelist, who was blinded to the first reviewer’s scores. This enabled the calculation of inter-rater reliability metrics and helped identify any systematic inconsistencies. The evaluators performed their reviews using a secure online portal where the anonymized records were assigned. They were instructed to rely primarily on Kenya Ministry of Health-approved clinical guidelines and related diagnostic and treatment protocols (which were made available via a reference repository) and their clinical judgement when assessing the appropriateness of the AI’s suggestions. In determining whether a clinician change was attributable to the LLM, evaluators classified a revision as LLM-induced only if it aligned with specific content in the LLM’s output and diverged from the initial documentation. Any changes that were inconsistent with, or unrelated to, the LLM’s suggestions were classified separately. They were not permitted to discuss cases with each other to avoid influence, but they could query the study coordinators or investigators if any patient record data was unclear.

For any case where the AI’s advice included potentially harmful elements, defined in Supplementary Material 2 as incorrect diagnoses, omitted critical differentials, inappropriate medication or dosing, inappropriate investigations, culturally/contextually inappropriate guidance or other reviewer-identified safety concerns, the evaluator documented this finding. In such instances, an additional review was performed: the patient’s follow-up records and any safety incident reports were examined (by the Penda Health clinical quality team) to determine if the clinician had actually acted upon the questionable AI recommendation and whether it led to any adverse outcome. Any such events were then managed in accordance with Penda Health’s standard operating procedures for clinical safety events, and reporting was deemed to be beyond the scope of this study.

### Outcomes and measures

The primary outcomes of interest were:

1. The quality of the initial documentation;
2. The appropriateness of any diagnostic reasoning;
3. The appropriateness of any clinical reasoning (including proposed investigations and treatment planning); and
4. The safety of the LLM’s recommendations, and whether both potentially harmful and beneficial recommendations appeared to be acted upon.

Results from the questions aligned with the above outcomes were summarized. For cases with identified unsafe advice, we recorded the nature of the risk (for example, misdiagnosis, incorrect medication and so on) and whether any actual patient harm occurred (as indicated by follow-up data).

### Statistical analysis

We used descriptive statistics to summarize the data. For reporting purposes, several rubric categories (Supplementary Material 2) were collapsed: (1) ‘Yes’ and ‘Somewhat’ were combined; (2) both ‘No’ categories were combined; (3) major and minor safety concerns were grouped together; (4) full and partial alignment categories were combined; and (5) full and partial mitigation categories were similarly combined. Variables were reported as counts and percentages. We calculated exact binomial 95% CIs for all proportions of the evaluation metrics across the quality and safety domains. Interrater reliability was assessed on a subset of cases that were duplicated and independently reviewed by two evaluators. For ordinal outcomes, including the quality of initial clinical documentation, whether the LLM addressed identified issues, alignment with patient context, clinician modification of documentation, and reduction of identified risks, we calculated Kendall’s *W* statistic to quantify agreement among reviewers. For binary and nominal outcomes, including the presence of unsafe advice, hallucinations, evidence of diagnostic reasoning, and provision of a reasonable differential diagnosis, we calculated Fleiss’ kappa. Agreement strength was interpreted using the Landis and Koch benchmarks^38^. All analyses were conducted using R (version 4.5.1).

### Patient and public involvement statement

Patients were not directly involved as research participants.

### Ethics and inclusion

This study was conducted in accordance with international and national ethical guidelines for research. The protocol was reviewed and approved by the Amref Ethics and Scientific Review Committee (ESRC) in Kenya (P1839-2025). Given that the study involved the secondary use of de-identified clinical data and posed minimal risk to patients, the ethics committee granted a waiver of informed consent. To protect patient confidentiality, all data were anonymized before analysis, and no personal identifiers appear in any reports or outputs. The evaluation panel members signed confidentiality agreements and accessed the records through a secure platform. All procedures adhered to the principles of the Declaration of Helsinki and Good Clinical Practice. The study was codesigned and implemented with local researchers, who were actively involved in study design, data collection, evaluation, interpretation and authorship. The evaluation panel comprised locally licensed Kenyan physicians. Findings were shared with local stakeholders to support service improvement and to ensure the research remained relevant and beneficial to the communities served.

### Reporting summary

Further information on research design is available in the Nature Portfolio Reporting Summary linked to this article.

## Supplementary information

Supplementary Information Supplementary Material 1 (Supplementary Box 1, Tables 1–6 and Fig. 1), Material 2 and Material 3.

Reporting Summary

## Data Availability

To enable replication of the study results, the full set of expert panel evaluation data and the analysis code have been deposited and are openly available via Zenodo at 10.5281/zenodo.17107852 (ref. ^39^). Clinical data from patient records are subject to approvals from a local ethics review board, as well as agreement with the clinical partner (Penda Health), in line with best practices for the unconsented reuse of routine data.

## Code availability

No custom code was used for data collection. Analysis scripts are openly available at our GitHub repository and via Zenodo at 10.5281/zenodo.17107852 (ref. ^39^).
