# Supplementary material for: Safety of a large language model-based clinical decision support system in African primary healthcare
Source: Nat Health. 2026 Mar 10;1(6):607–18. doi: 10.1038/s44360-026-00082-5 (PMC13246507; doi:10.1038/s44360-026-00082-5)
Supplement: Supplementary file 2 — Reporting Summary [file 44360_2026_82_MOESM2_ESM.pdf]

nature portfolio

Corresponding author(s): Bilal A. Mateen

Last updated by author(s): Feb 8, 2026

Reporting Summary

Nature Portfolio wishes to improve the reproducibility of the work that we publish. This form provides structure for consistency and transparency in reporting. For further information on Nature Portfolio policies, see our Editorial Policies and the Editorial Policy Checklist.

Statistics

For all statistical analyses, confirm that the following items are present in the figure legend, table legend, main text, or Methods section.

n/a	Confirmed	
☐	☒	The exact sample size (*n*) for each experimental group/condition, given as a discrete number and unit of measurement
☐	☒	A statement on whether measurements were taken from distinct samples or whether the same sample was measured repeatedly
☐	☒	The statistical test(s) used AND whether they are one- or two-sided<br>*Only common tests should be described solely by name; describe more complex techniques in the Methods section.*
☐	☒	A description of all covariates tested
☐	☒	A description of any assumptions or corrections, such as tests of normality and adjustment for multiple comparisons
☐	☒	A full description of the statistical parameters including central tendency (e.g. means) or other basic estimates (e.g. regression coefficient) AND variation (e.g. standard deviation) or associated estimates of uncertainty (e.g. confidence intervals)
☐	☒	For null hypothesis testing, the test statistic (e.g. F, t, r) with confidence intervals, effect sizes, degrees of freedom and P value noted<br>*Give P values as exact values whenever suitable.*
☒	☐	For Bayesian analysis, information on the choice of priors and Markov chain Monte Carlo settings
☒	☐	For hierarchical and complex designs, identification of the appropriate level for tests and full reporting of outcomes
☒	☐	Estimates of effect sizes (e.g. Cohen's *d*, Pearson's *r*), indicating how they were calculated

Our web collection on statistics for biologists contains articles on many of the points above.

Software and code

Policy information about availability of computer code

Data collection	No custom code was used for data collection.
Data analysis	All statistical analyses were conducted using R (version 4.5.1). Analysis scripts are openly available at our GitHub repository and archived on Zenodo: https://doi.org/10.5281/zenodo.17107852.

For manuscripts utilizing custom algorithms or software that are central to the research but not yet described in published literature, software must be made available to editors and reviewers. We strongly encourage code deposition in a community repository (e.g. GitHub). See the Nature Portfolio guidelines for submitting code & software for further information.

Data

Policy information about availability of data

All manuscripts must include a data availability statement. This statement should provide the following information, where applicable:

- Accession codes, unique identifiers, or web links for publicly available datasets
- A description of any restrictions on data availability
- For clinical datasets or third party data, please ensure that the statement adheres to our policy

Data originated from routine electronic medical record (EMR) entry by clinicians at 16 Penda Health facilities. For study evaluation, an independent panel of trained local physicians reviewed and annotated encounters using a standardized rubric. To enable replication of the study results, the full set of expert panel evaluation data and the analysis code have been deposited and are openly available on Zenodo: https://doi.org/10.5281/zenodo.17107852. Clinical data from patient records

are subject to approvals from a local ethics review board, as well as agreement with the clinical partner (Penda Health), in line with best practices for the unconsented reuse of routine data.

Research involving human participants, their data, or biological material

Policy information about studies with human participants or human data. See also policy information about sex, gender (identity/presentation), and sexual orientation and race, ethnicity and racism.

Reporting on sex and gender	Sex (male/female) was routinely recorded in the EMR and included in the study dataset. Analyses were not stratified by sex or gender, as this was not directly relevant to the study objectives.
Reporting on race, ethnicity, or other socially relevant groupings	No data on race, ethnicity, or other socially constructed groupings were collected or analysed.
Population characteristics	The study included 1,469 de-identified patient encounters sampled from routine primary care consultations. Approximately 24% of patients were aged 0-4 years (n=350), 15% were 5-17 years (n=225), 50% were 18-39 years (n=730), 10% were 40-59 years (n=150), and 1% were ≥60 years (n=14). Fifty-eight percent were female (n=857) and 42% male (n=612). Clinical presentations were predominantly respiratory (38%), gastrointestinal (25%), and genitourinary/reproductive (15%), with smaller proportions dermatological, musculoskeletal, febrile/infectious, neurological/psychiatric, and other categories. Documentation quality and safety-related clinical variables were also assessed. Data were derived from routinely collected, de-identified electronic medical records. No genotypic or biomarker information was collected or analysed.
Recruitment	This was a retrospective analysis of routine clinical encounters. No active patient recruitment was undertaken. Patients had the option to opt out of secondary use of their data under Penda Health's institutional policy.
Ethics oversight	The study was reviewed and approved by the Amref Ethics and Scientific Review Committee (ESRC) in Kenya (protocol P1839-2025). A waiver of informed consent was granted given the secondary use of de-identified clinical data and the minimal risk posed to patients

Note that full information on the approval of the study protocol must also be provided in the manuscript.

Field-specific reporting

Please select the one below that is the best fit for your research. If you are not sure, read the appropriate sections before making your selection.

☒ Life sciences ☐ Behavioural & social sciences ☐ Ecological, evolutionary & environmental sciences

For a reference copy of the document with all sections, see nature.com/documents/nr-reporting-summary-flat.pdf

Life sciences study design

All studies must disclose on these points even when the disclosure is negative.

Sample size	We drew a stratified random sample of 1,500 encounters from Penda Health's EMR (strata by age). This size was chosen pragmatically to balance reviewer workload with precision and subgroup coverage, and is sufficient to estimate proportions as low as 2% with ±1% precision, accounting for a design effect of 2.
Data exclusions	We excluded (i) encounters where the AI Consult was not used (inclusion required the comprehensive/summarized prompt; only Comprehensive Consult encounters were analysed), (ii) non-clinical visits that typically do not invoke the AI Consult (e.g., routine immunizations, family-planning, drug refills), and (iii) records with a documented patient opt-out; opt-out records were replaced by randomly sampled encounters from the same age stratum.
Replication	Reproducibility was assessed through duplicate blinded review of 10% of encounters. Agreement varied across domains. Most ordinal ratings demonstrated fair to moderate concordance (Kendall's W 0.27–0.47), with substantial agreement for clinician-modified documentation ($W = 0.648$, $p < 0.001$). Binary indicators exhibited lower κ values (−0.007–0.114), consistent with the known κ paradox under extreme prevalence, where high observed agreement can yield low. Detailed results for all metrics are provided in Supplementary Table S1.
Randomization	This was a retrospective observational study with no allocation to interventions. Encounters were selected using stratified random sampling by age group from the EMR. Analyses were descriptive in nature (e.g., proportions, medians, inter-rater reliability metrics), rather than comparative across groups.
Blinding	For the duplicate-review subset, evaluators were blinded to each other's scores and instructed not to confer; blinding to patient characteristics was not possible as these details are integral to the records under review.

Reporting for specific materials, systems and methods

We require information from authors about some types of materials, experimental systems and methods used in many studies. Here, indicate whether each material, system or method listed is relevant to your study. If you are not sure if a list item applies to your research, read the appropriate section before selecting a response.

Materials & experimental systems

n/a	Involved in the study
☒	☐ Antibodies
☒	☐ Eukaryotic cell lines
☒	☐ Palaeontology and archaeology
☒	☐ Animals and other organisms
☒	☐ Clinical data
☒	☐ Dual use research of concern
☒	☐ Plants

Methods

n/a	Involved in the study
☒	☐ ChIP-seq
☒	☐ Flow cytometry
☒	☐ MRI-based neuroimaging

Plants

Seed stocks	Not applicable
Novel plant genotypes	Not applicable
Authentication	Not applicable
